# Text mining for neuroanatomy using WhiteText with an updated corpus and a new web application

**DOI:** 10.3389/fninf.2015.00013

**Published:** 2015-05-21

**Authors:** Leon French, Po Liu, Olivia Marais, Tianna Koreman, Lucia Tseng, Artemis Lai, Paul Pavlidis

**Affiliations:** ^1^Rotman Research Institute, University of TorontoToronto, ON, Canada; ^2^Department of Psychiatry, University of British ColumbiaVancouver, BC, Canada; ^3^Centre for High-Throughput Biology, University of British ColumbiaVancouver, BC, Canada

**Keywords:** connectome, text mining, natural language processing, information retrieval

## Abstract

We describe the WhiteText project, and its progress towards automatically extracting statements of neuroanatomical connectivity from text. We review progress to date on the three main steps of the project: recognition of brain region mentions, standardization of brain region mentions to neuroanatomical nomenclature, and connectivity statement extraction. We further describe a new version of our manually curated corpus that adds 2,111 connectivity statements from 1,828 additional abstracts. Cross-validation classification within the new corpus replicates results on our original corpus, recalling 67% of connectivity statements at 51% precision. The resulting merged corpus provides 5,208 connectivity statements that can be used to seed species-specific connectivity matrices and to better train automated techniques. Finally, we present a new web application that allows fast interactive browsing of the over 70,000 sentences indexed by the system, as a tool for accessing the data and assisting in further curation. Software and data are freely available at http://www.chibi.ubc.ca/WhiteText/.

## Introduction

Neuroinformatics research thrives on plentiful amounts of open and computable neuroscience datasets. This type of data is lacking at the level of brain regions, when compared to molecular data about genes or proteins. Currently, the bulk of this neuroscience information is fragmented across the literature. Manual curation can join and formalize the findings (Bota et al., 2012, 2014). To speed up this process in the domain of anatomical connectivity, we created the WhiteText project to automatically extract this information from text. WhiteText was designed to extract mentions of brain regions and statements describing connections between them. While developing WhiteText we asked the following questions:

1. How accurately can neuroanatomical connectivity information be automatically extracted from neuroscience literature?
2. What textual features are useful for automatic extraction?
3. How much connectivity data is available in neuroscience abstracts?
4. Which species are used for connectivity studies?

By extracting thousands of connectivity statements and addressing these questions, we were able to evaluate several text processing techniques and create neuroinformatics resources for connectivity knowledge.

Connectomics is of great interest to neuroscientists that seek to understand brain networks using complete connectivity maps. Global connectivity knowledge is often viewed as fundamental to understanding how the brain processes information. Local connectivity informs focused studies involving smaller numbers of brain regions. Several large-scale projects are seeking to uncover brain region level connectivity maps in human, macaque, rat and mouse. Using advanced magnetic resonance imaging, the Human Connectome Project will provide brain region level connectivity maps for 1,200 healthy individuals to understand human neuroanatomy and its variation (Van Essen et al., 2013). Using neural tracers, the Mouse Connectome Project (Zingg et al., 2014), the Mouse Brain Architecture Project (brainarchitecture.org) and Allen Mouse Brain Connectivity Atlas (Oh et al., 2014) provide connectome scale data obtained from standardized approaches. The Allen Mouse Brain Connectivity Atlas has produced the most complete and standardized mouse connectome which covers 295 disjoint regions (Oh et al., 2014). The preceding Brain Architecture Management System (BAMS2) contrasts these experimental efforts by providing curated reports of rat brain connectivity (Bota et al., 2014, 2015). The BAMS curators standardize published connectivity results obtained from independent labs into a single database. CoCoMac is a similar system that collates connectivity results from Macaque studies (Stephan, 2013). The WhiteText project seeks to complement these projects by automatically extracting connectivity reports from past studies to speed up manual curation and add context to large-scale connectome projects.

**Figure 1:**
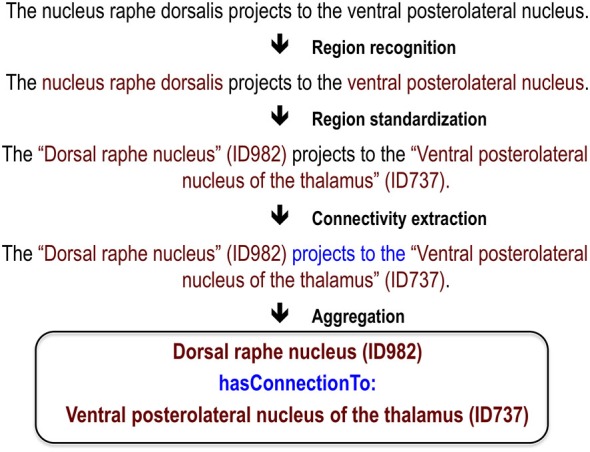
**Visualization of processing steps for an example sentence**.

While there has been significant effort to mine neuroscience information from text (Ambert and Cohen, 2012), our work is most inspired by past efforts to extract information about protein-protein interactions. This task is analogous to extracting connectivity information: it requires extraction of interaction relationships between named entities (proteins instead of brain regions). This close analogy allowed us to leverage work done in the gene and protein domain. At the BioCreative III workshop challenge, 23 teams competed to extract, resolve and link protein and gene mentions (Arighi et al., 2011), generating information on effective approaches. We adapted and extended the text-mining methods previously used to analyze protein networks for extraction of connectivity between brain regions (Tikk et al., 2010). This is an attractive approach because many of the challenges in analyzing text for information about proteins are also faced in mining information about brain regions. These challenges include abbreviations, synonyms, lexical variation and ambiguity. A key foundation for both the BioCreative challenges and our work are hand-annotated corpora to use as training data and gold standards for evaluation. Here we first review our annotated corpus and the methods and results for three tasks required for extracting connectivity relationships between brain region pairs: recognition of brain region mentions, standardization of brain region mentions and connectivity statement extraction (Figure 1). We provide only summary results and methods for these tasks and refer readers to the corresponding publications for details. Then we describe a recent evaluation which we used to create a new corpus; and finally a website we created to view the results.

## Review of Progress

### Manually Annotated Corpus

To seed the project we annotated a set of 1,377 abstracts for brain region mentions and connectivity relations (French et al., 2009). We used abstracts rather than full-text documents due to accessibility and their higher proportion of summary statements. This initial corpus allows for training of machine learning methods and later comparison between automatic and manually derived annotations. We focused on abstracts from one journal, the Journal of Comparative Neurology (JCN), because it is enriched with neuroanatomical studies. As described below, we considered other journals in later analyses.

Two trained undergraduate research assistants annotated the corpus for brain region mentions and connectivity relations in any species. Annotated brain region spans matched for 90.7% of the mentions in the subset of 231 abstracts annotated by both curators. In total, 17,585 brain region mentions were annotated with a subset forming 4,276 connections. Three high accuracy text mining methods were applied to all abstracts: species recognition (Gerner et al., 2010), automated expansion of abbreviations (Schwartz and Hearst, 2003) and tokenization (McCallum, 2002; Cunningham et al., 2013). Abbreviations were expanded because they are a common source of ambiguity and confusion (Gaudan et al., 2005). Further, we processed text within individual sentences that would not contain abbreviation expansion information found in previous sentences. Rat was the most common species of the 209 species studied (aside from species relating to reagents used in tract tracing such as horseradish, wheat and pseudorabies virus).

### Recognition of Brain Region Mentions

The first task of recognizing mentions of brain regions in free text is known as “named entity recognition”. This step identifies (“highlights”) spans of text that refer to brain regions (a “mention”). For this task we employed the MALLET package for natural language processing (McCallum, 2002) to create a conditional random field classifier that was able to label brain region mentions (French et al., 2009). Eight-fold cross-validation was used in this evaluation and abstracts were not split between training and testing. In this cross-validation framework each sentence is used seven times for training and once to test. Feature selection was performed with 14% of abstracts held-out. We consistently define precision as the proportion of true positives to positive predictions and recall as the proportion of true positives to actual positives. For this task the classifier recalled 76% of brain region mentions at 81% precision. Precision increases to 92% and recall reaches 86% when partial matches are counted. This performance was much higher than naive dictionary-based methods that attempt to match words to lists of known brain region names. We observed that regions in non-mammals (e.g., insects), which were underrepresented in the corpus, were poorly classified. Thus recall improves when restricting the abstracts to studies of monkey, cat, rat and mouse brain but only in comparison to a similar sized set of random abstracts.

From our analysis, we suspect many incorrectly recognized brain region mentions are due to conjunctions, previously unseen words and brain regions of less commonly studied organisms (e.g., insects and fish). Surrounding words, word base forms and abbreviation expansion were the most informative techniques and features used by the classifier. Although textual features derived from the neuroscience domain did increase performance (lexicons of brain region names for example), we found that most of the knowledge needed to extract brain region mentions can be learned from our large set of annotated examples.

### Standardization

Recognition of brain region mentions only provides a string that is predicted to refer to a brain region. Standardizing this string to a formally defined concept representing the brain region is important to downstream analysis and linking to other resources. The process of mapping free text to formal identifiers is also known as normalization or resolution. For example, this step aims to link the free text “substantia nigra compact part” or “SNC” found in an abstract to the NeuroLex concept birnlex_990 which has the preferred name of “Substantia nigra pars compacta” (Bug et al., 2008). Viewing birnlex_990 in the NeuroLex website expands the mention to a definition, information about spatial location and cell types found in the region (Larson and Martone, 2013). To maximize our set of brain region names, we targeted five neuroanatomical lexicons that span several species [NeuroNames (Bowden et al., 2011), NIFSTD (Imam et al., 2012), the Brede Database (Nielsen, 2015), BAMS (Bota et al., 2014), and the Allen Mouse Brain Reference Atlas (Dong, 2008)]. This provided a set of 11,909 target region names that represent an estimated 1,000 different mammalian brain regions (French and Pavlidis, 2012).

For the standardization task we applied simple lexicon-based methods that iteratively modified the original mention until a match was found (French and Pavlidis, 2012). First, a case-insensitive exact string match was attempted on the mentioned region. If that failed to match, word order was ignored by using bag-of-words matching, so that “reticular thalamic nucleus” would match “thalamic reticular nucleus”. Next, stemming was applied to reduce words to base forms (e.g., “nucleus raphé dorsalis” would reduce to “nucleu raph dorsali”). Again, exact matching of stemmed mentions and bag-of-stems matching was attempted. These methods were stringent, as they required all words or stems in a mention to match the name in the lexicons. To improve coverage we designed twelve modifiers that edited the mentions, sacrificing some information. This included removing hemisphere specific qualifiers, bracketed text and directional prefixes. Application of these modifiers increased standardization coverage from 47–63%.

By testing the above approaches on the manually annotated corpus, we estimated that mentions are mapped at 95% precision and 63% recall (French and Pavlidis, 2012). We note that precision is based on the lexical information (brain region names) and not the specific neuroanatomical location in a given species and atlas. This step is key for many neuroscience text miners because it provides a method for linking abstracts to region-specific data outside the text via formalized brain region names. In addition, patterns of publication interest can be observed: not surprisingly, some regions are more popular than others but popularity can wax and wane over time. Importantly, this work quantified challenges in the standardization of neuroanatomical nomenclatures. We observed that many standardized terms never appear in our input corpus and many mentions used by authors are not in the terminologies. To address this latter gap, we deposited 136 brain region names identified from our analysis into the Neuroscience Lexicon (Larson and Martone, 2013).

### Connectivity Statement Extraction

In a given abstract, mentions of brain regions provide limited information without any context. Our goal was to extract information about the brain regions, namely connectivity. To reduce the complexity of this task we targeted positive statements of connectivity and ignore the direction (efferent/afferent). Further we limited the manually curated training and test connections to those within sentences. These restrictions allow application of existing tools for extracting protein-protein interactions. The resulting classification task is to determine if a pair of brain region mentions are described as connected or not. A negative prediction includes statements reporting no connectivity between the two regions but the majority of negative pairings are from sentences mentioning two brain regions but containing no connectivity information. Our corpus had available 22937 total pairs of brain regions of which 3097 describe connections, with the balance considered negative examples (French et al., 2012).

By re-using the protein interaction benchmark tools assembled by Tikk and colleagues, we tested several methods on our annotated corpus in a cross-validation framework (Tikk et al., 2010). The best method, the “shallow linguistic kernel” (Giuliano et al., 2006) recalled 70% of the sentence level connectivity statements at 50% precision in ten-fold cross-validation. This method is “shallow” in the sense that it does not involve parsing complex sentence structure. Sentence length does increase from the top to bottom ranked predictions, suggesting a relationship with complexity. However, similar accuracy was provided by more complex methods that use deeper features such as word dependencies and semantic features.

## Methods

### Extended Evaluation and Corpus Creation

Each interaction was independently judged by two of four undergraduate research assistants and disagreements were resolved by group review. All four curators annotated a small training set of 307 connections for initial training and guideline refinement. Annotator agreement depended on the pair of curators compared, and ranged between 83% and 97%. To speed curation, we used spreadsheets that presented the full sentences and links to the abstracts containing the predicted connections.

### Article Classification and Expanded Predictions of Connectivity

The online MScanner tool was used to find connectivity abstracts outside of the JCN (Poulter et al., 2008). MScanner is not domain specific, but instead uses supervised learning to search PubMed for related articles (Naïve Bayes classifier). Abstracts found to contain connectivity statements in previous evaluations were used as the input set. We applied MScanner with and without the word features (journal name and MeSH terms features were used for both executions). Brain region mentions were extracted with the previously published conditional random field that was trained on the entire first set of manually annotated abstracts. The shallow linguistic kernel (Giuliano et al., 2006) from the ppi-benchmark framework was used to predict connectivity relations (Tikk et al., 2010).

### WhiteText Web

WhiteText Web was implemented with Google Web Toolkit 2.5.1 and the Apache Jena framework. User input is restricted to Neurolex brain regions that appear in the corpus. We note that this restriction is only placed on the one of the two connected regions, allowing any brain region mention text to represent the second region displayed (“Connected Region”). Formalized mapping to synonyms and query expansion to subregions was extracted from the Neuroscience Information Framework (NIF) Gross Anatomy ontology. Subregions were extracted by extracting “proper_part_of” predicates [Open Biomedical Ontologies (OBO) and relation ontology (RO)]. The list of 110 phrases that describe connectivity are that underlined in the output were extracted from the first manually annotated corpus. Example phrases are “projects to” and “terminating in” (full list on supplementary website). LINNAEUS was used to recognize and normalize species names (Gerner et al., 2010). Sentences from abstracts with more than one species mentioned are duplicated to prevent omission of connections when sorting the table by species.

## Results

### Extended Evaluation and Corpus Creation

Beyond the cross-validation evaluation described above, we have previously applied our method to 12,557 previously unseen JCN abstracts (those not in our corpus) and compared a standardized subset of 2,688 relationships to the data in BAMS (Bota et al., 2012). We found that 63.5% of these connections were reported in BAMS. Using the BAMS data as a gold standard, we also found that precision can be increased at the cost of recall by requiring connections to occur more than once across the corpus (French et al., 2012).

**Figure 2:**
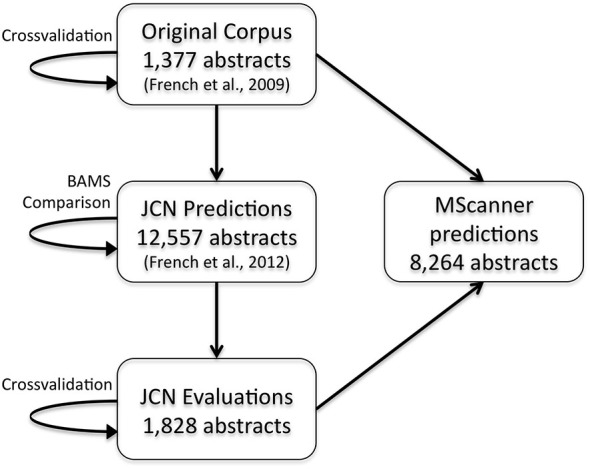
**Flow chart depicting the origins and evaluations of the connectivity corpora**. Arrows represent the use of annotated data from one corpus (source) to test or create a corpus (target). JCN, Journal of Comparative Neurology; BAMS, Brain Architecture Management System.

To extend these results and obtain more training data, we have now created a new corpus by extending our previous evaluation of 2000 positive predictions (French et al., 2012). Figure 2 outlines the creation of new corpora from the original corpus. This new corpus is based on running our framework on the test set of 12,557 JCN abstracts. Most importantly, to gauge recall we had to identify negative examples, as our previous effort only manually evaluated positive predictions. By adding new evaluations of negative predictions, the new corpus contains 11,825 brain region pairings extracted from the 12,557 abstracts (12% of possible within sentence pairings), of which 18% were considered positive examples. Recall was 45.5% (as previously reported on the 2000 positive predictions, precision is 55.3%). The drop in accuracy compared to the previous cross-validation test appears to be partly due to automation of preprocessing steps that were done manually in the original corpus of 1,377 abstracts. These automated steps are imperfect and thus a source of errors upstream of the connection prediction step. Specifically, we found that many classification errors could be ascribed to problems with brain region mention extraction (~10–15% of errors) and abbreviation expansion (<4%). Standardization of brain region mentions was not performed in this evaluation, allowing isolation from low recall in the standardization task. In a cross-validation framework, the shallow linguistic kernel within this new set of evaluated connections replicates the accuracy of the first set (recalling 67% of connectivity statements at 51% precision). In comparison to our first set, this corpus covers a broader set of abstracts but a lower number of connections (Table 1). We are making this corpus available to the community to use in further efforts at improving connectivity extraction methods or for developing other text processing tasks that benefit from the annotations provided.

**Table 1:** **Summary connectivity statistics for curated and predicted corpora**.

	Original corpus (French et al., 2009)	JCN evaluations	JCN predictions (French et al., 2012)	MScanner
Abstracts	1377	1828	12557	8264
Source	Curation	Evaluations	Classification	Classification
Region annotations	Manual	Automatic	Automatic	Automatic
Region pairs	22577	11825	156741	164555
Connections	3097 (16%)	2111 (18%)	28107 (22%)	36566 (22%)
Recall	70%	67%	NA	NA
Precision	50%	51%	NA	NA

*This table presents summary counts of abstracts and sentence level connectivity counts for abstracts with predicted and curated connections. Region pairs and connection counts are counted within sentences only. Connections were predicted with a shallow linguistic kernel trained on the Original Corpus for both the JCN Predictions (Journal of Comparative Neurology) and MScanner sets. Precision and recall values were computed with shallow linguistic kernel in a crossvalidation framework*.

### Article Classification and Expanded Predictions of Connectivity

In our initial work we focused on the Journal of Comparative of Neurology due to its enrichment of connectivity reports. Although it is possible to run our pipeline on the entire MEDLINE corpus, we suspected this would produce a large number of false positives from the enormous number of abstracts that mention brain regions but do not discuss connectivity. To create a larger connectivity resource from abstracts more likely to be relevant, we used the MScanner tool (Poulter et al., 2008). MScanner trains a classifier to identify abstracts with features similar to an input training set. Features used by MScanner include journal name, MeSH terms and words in the abstract and title. We trained MScanner using the abstracts from our curated corpora, reasoning that the results would be enriched for abstracts containing connectivity statements (Figure 2). The features chosen by MScanner as relevant include the word features “Nucleus”, “Medial”, “Projection” and the MeSH qualifier “anatomy and histology”. MScanner yielded a new set of 8,264 abstracts. Applying our pipeline to this set yielded 36,566 predicted statements of connectivity. Over 92% of abstracts were predicted to have at least one connectivity statement. This suggests MScanner provides a good initial filtering step for our pipeline. Beyond the general purpose approach of MScanner, Ambert and Cohen demonstrate more complex article classification tools for extraction of connectivity studies (Ambert and Cohen, 2012).

### WhiteText Web

**Figure 3:**
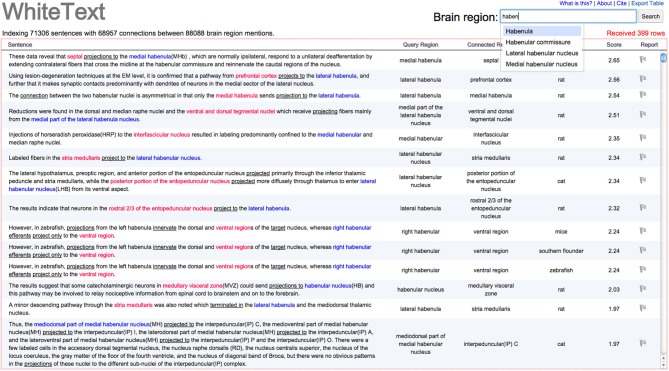
**Screenshot of example results from WhiteText Web**. The top text input field attempts to match typed text to brain regions in NIFSTD while the user types. The query region column shows the original named brain regions that were matched to the given input of “Habenula” or it’s children. Sentence text is directly linked to the source abstract in PubMed. Query and connected regions are colored, with underlines marking words that suggest connectivity. Results can be sorted by all columns except the first. A single click on the gray flag in the “Report” column allows users to mark sentences that were incorrectly parsed. The “Export Table” link (top left) provides a tab-separated file containing the returned results.

We created WhiteText Web,^1^ to provide easy access to the extracted connectivity statements. Given an input brain region, the tool returns all predicted statements of connectivity involving that region and its enclosing subregions. The resulting sentences and connections are highlighted for quick browsing by the user. As shown in Figure 3, results are provided in a spreadsheet table format that allows quick sorting by classification score (approximates prediction confidence), connected regions and species mentioned in the abstract. Source abstracts are easily accessible to allow review of full context. Each predicted connectivity relation presented is displayed with a flag icon, which allows a user to flag connections that appear incorrect. This user provided information is logged and will be used in future evaluations.

### Characterization of WhiteText Web Combined Corpus

**Figure 4:**
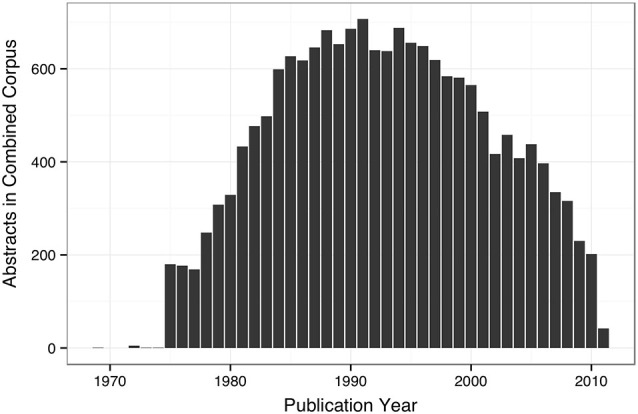
**Bar plot of yearly counts of abstracts with connectivity information in the combined corpus**.

The WhiteText Web corpus consists of all the curated and predicted connectivity statements mentioned above (17,454 abstracts with at least one connection). Over 200 species were mentioned in these abstracts with rat (24,690 predicted connections), cat (12,469) and rhesus macaque (3,113) having the most mentions (top ten shown in Table 2). The JCN is still the main source for our connectivity information due to its selection for the original corpora. However, the use of MScanner has provided abstracts with connectivity predictions from 304 additional journals with the top ten given in Table 3. Publication year in the combined corpus is limited by available abstracts in the MEDLINE database (8 abstracts found before 1,975) and the dates of our studies and the MScanner database (no abstracts beyond mid 2011). Yearly counts of connectivity studies contained in our combined corpus peaks in 1991 with 707 abstracts (Figure 4).

**Table 2:** **Species with the most associated connections in the combined corpus**.

Species name	NCBI species identifier	Connections
Rattus norvegicus	10116	24690
Cat	9685	12469
Rhesus monkey	9544	3113
Rat	10118	2368
Rabbit	9986	1497
Human	9606	1258
Macaca fascicularis	9541	1218
Mouse	10090	1107
Chiecken	9031	728
Guinea-pig	10141	611

*Connection counts combine predicted and curated connections in the corpus. NCBI taxonomy identifiers are provided*.

**Table 3:** **Top ten most frequent journals in the combined corpus**.

Journal name	Abstracts
The Journal of comparative neurology	9815
Brain Research	1643
Neuroscience	938
Experimental brain research	627
The Journal of neuroscience	369
Brain research bulletin	365
Neuroscience letters	326
Brain, behavior and evolution	251
Anatomy and embryology	231
The European journal of neuroscience	207

### Accessibility

Data and software used for the project are freely available in standardized formats.^2^ The new corpus is additionally provided at http://figshare.com/articles/New_WhiteText_Corpus/1400541. Text mined results for a specific brain region specific can be exported from WhiteText Web as a tab separated files. To store annotated text we used GATE and encoded downstream annotations in AirolaXML and Resource Description Framework (RDF). Use of RDF allows simple queries of extracted connectivity statements with the SPARQL query language. Connectivity matrices are also provided for convenience. Software and documentation is available on GitHub.^3^ Further, Bluima, an open source text mining toolkit for neuroscience has re-used some of our resources for brain region extraction in the UIMA framework (Unstructured Information Management Architecture; Richardet and Telefont, 2013).

## Discussion

We created and applied a system for large-scale automatic extraction of connectivity knowledge. By analyzing over 20,000 abstracts we found the neuroscience literature contains a wide diversity of terms, species, and brain region names. Unfortunately, this diversity exceeds that of the existing formalized neuroanatomical lexicons. We found it difficult to create a clear set of annotation guidelines due to this diversity that extends to sentence structure and experiment design. While this diversity limits the automatic mining of neuroscience literature, we evaluated several methods that improve automatic extraction. We found great value in general-purpose and biomedical text mining tools. We applied these tools with little or no tuning and report robust and extendable results. This allowed more time for extensive manual evaluation and review. In addition to tested methods, our work provides a database of evaluated connectivity statements that can be used as a starting point for manual curation and to facilitate neuroscience text mining.

Our results and evaluations provide the most critical assessment of text mining for neuroscience to date. We note the NeuroScholar system which had similar overall goals to our project (Burns and Cheng, 2006). Burns and Cheng sought to automatically label more detailed information about connectivity experiments in full text articles, including methodological details. In contrast, our work focuses on summary statements in abstracts to extract brain region mentions and relationships between them. Building on the WhiteText project resources, a large-scale application of connectivity extraction has been performed on all PubMed abstracts and a large set of full text articles (Richardet et al., 2015). Richardet and colleagues used the WhiteText corpus to help develop a scalable system that combined the shallow linguistic kernel with filer and rule based methods. By using the Allen Mouse Brain Connectivity Atlas, precision of the extracted connections was estimated at 78%. Both studies support the value and feasibility of automatically extracting connectivity information from natural language text.

It is possible to search for connectivity literature using keyword searches of PubMed or Google Scholar. However, searches for a single brain region will retrieve studies of that region that do not examine connectivity. Adding keywords like “projections” will not recall all studies as demonstrated by our list of over 100 phrases that describe a connection. In contrast our system is focused on connectivity studies and only presents users with sentences predicted to contain connectivity statements. We designed WhiteText Web for neuroscientists searching connectivity studies that can be used to design or interpret their experiments. We also designed it to aid curation by adding an easy way to report incorrect predictions. The features of WhiteText Web are similar to NIF Integrated Nervous System Connectivity resource (Larson and Martone, 2013). Our text-mined results are less accurate than the NIF connectivity resource, which is based on six manually curated databases. Also, the NIF resource provides the direction of the connections and reports of no connectivity. However, WhiteText Web provides a wider search covering more species and sources and underlines key words that indicate direction (“projects to” and “terminated in” for example). Further, WhiteText Web provides original text with highlights for quick viewing and the ability to provide instant feedback. NIF and BAMS2 provide increasingly valuable resources for integration and validation as they continue to grow with the published literature (Bota et al., 2015).

Recently, two teams reported large-scale tract-tracing studies in mouse (Oh et al., 2014; Zingg et al., 2014). Our system can supplement these studies by providing evidence of connectivity in other species and providing literature context for the connections. Unlike the large-scale surveys, the connectivity statements we extract are often from studies that potentially provide additional context and relationships. For example, future work could extract behavior and systems that are related to a connection by examining the abstract or full text that contains the connection.

Our work has several limitations. First, our analysis was limited to article titles and abstracts. Application of our methods to the complete texts of papers should provide more brain region mentions that can be mined for connections and other relationships. However, a recent study of neuroscience document classification demonstrated the difficulty of using full text, reporting low performance when using information from full text compared to abstracts for an article classification task (Ambert et al., 2013). This is presumably because abstracts tend to be highly concentrated with factual statements about the study compared to the rest of the article. Like Cohen and colleagues we believe better tools may be needed to fully exploit the different content and structure of full text (Cohen et al., 2010). Richardet et al. address these questions by extracting connectivity from over 630,216 full text neuroscience articles to predict over 250,000 connectivity relations, more than what was extracted from all PubMed abstracts (Richardet et al., 2015). They found that specific filtering rules were needed for full text. However, they report no differences between connections extracted from full text and abstracts in terms of distance and precision. Another limitation is that our tools were limited to analysis of single sentences, so connections that are described in more than one sentence could not be captured. We estimate that at least 25% of connections mentioned in an abstract span multiple sentences, a substantial loss of information for the text mining approach. Directionality of connections is also lost as our methods only predict presence of connection. However, we note that directions are annotated in the first corpus and WhiteText Web underlines direction-specifying keywords for the user. Finally, the accuracy of text mining and natural language processing limits the immediate application and biological interpretation of the results without further manual curation. To further improve the quality of the data in WhiteText, we are performing manual curation to remove errors. The text mining approach can provide a large set of data that is highly enriched for relevant statements, providing a good input for manual curation.

## Conflict of Interest Statement

The authors declare that the research was conducted in the absence of any commercial or financial relationships that could be construed as a potential conflict of interest.

## Footnotes

^1^http://www.chibi.ubc.ca/whitetext/app/

^2^http://www.chibi.ubc.ca/WhiteText

^3^https://github.com/leonfrench/public/wiki
